# Ten quick tips for staying safe online

**DOI:** 10.1371/journal.pcbi.1008563

**Published:** 2021-03-03

**Authors:** Danielle Smalls, Greg Wilson

**Affiliations:** 1 MIR Community Group, Raleigh, North Carolina, United States of America; 2 RStudio PBC, Boston, Massachusetts, United States of America; Dassault Systemes BIOVIA, UNITED STATES

## Introduction

Researchers studying everything from sexual health to the Coronavirus Disease 2019 (COVID-19) to gun violence are increasingly likely to be targeted because of their work. While research institutions have rules and guidelines for safeguarding sensitive information, these usually do not address the problem of keeping individuals safe from either targeted attacks like Climategate [1] or the kinds of “drive-by” threats that everyone now faces regardless of their occupation.

Hollywood depictions of everyday threats are as far from reality as their portrayals of scientists, but more realistic guidance for personal digital security is now freely available [2–4]. The 10 quick tips in this paper are a starting point: While they apply to everyone, they were developed with researchers in mind. While researchers expect their work to be scrutinized by the academic community, they should not expect to endure harassment due to the visibility of their published works. These rules do not guarantee complete safety, any more than seatbelts guarantee safe driving, but following them greatly reduces the likelihood of harm.

### Rule 1: Put on your own mask

The first and most important rule is that we should not rely on companies, universities, and other institutions to protect us, for the simple reason that they are not penalized if they don’t. As recently as 10 years ago, we could blame the lack of meaningful institutional liability for data breaches on the law being slow to catch up with rapidly changing technology. Accountability for these breaches is practically nonexistent: Data breaches have minimal impact on companies’ profitability, and individuals are almost never fined, much less jailed.

Much of what institutions force us to go through is **security theater** intended to make us believe something is being done rather than to actually make us safer. Requiring people to take off their shoes at airports is 1 example; random searches of backpacks and bags at the entrance to the subway is another, since it’s hard to imagine that a would-be attacker wouldn’t just go to another entrance. (Bruce Schneier’s blog [5] has many examples of security theater and the harm it does.)

Security theater is counterproductive because it encourages us to cut corners in ways that actually make us less safe. For example, forcing people to change passwords every 3 months encourages people to choose memorable (and therefore easy-to-guess) passwords.

### Rule 2: Digital security is rarely the weakest link

The second rule is to remember that most attacks take place offline and that the most effective ones are often the simplest. At an airport several years ago, 1 author heard a professor of computer science try to reset an online account over the phone. In just a couple of minutes, they had inadvertently told everyone in the lounge their full name, their date of birth, the 3-digit verification code on the back of their credit card, and what was almost certainly their mother’s maiden name.

The moral of this story is that safety comes from good habits, not technology. **Social engineering** is far more common than hacking: In practice, it is far easier to trick someone into giving you their password than to break into their devices digitally.

The key practice is **situational awareness**, which is a fancy way of saying, “Pay attention to what’s happening and respond accordingly.” If you start working on a high-profile subject that will likely attract controversy, you should take more precautions than usual. For example, someone should recognize that agreeing to be an expert witness increases the odds that they will be targeted and should be more careful about what he puts into email while preparing and delivering his testimony.

The corollary to situational awareness is to de-escalate when you can. Being on guard all the time is exhausting and quickly leads to **security fatigue** [6]. If you are too tired to follow good practices, knowing them does you no good.

Two technologies that are useful, but only to a certain extent, are virtual private networks (VPNs) and a specialized web browser called “Tor.” A VPN connects your device to a server, then has the server make connections to other machines on your behalf. All messages between your device and the server are encrypted, and the server can be managed by professional IT staff in a jurisdiction with tight privacy laws to increase your safety. Tor routes messages randomly through a network of servers, making traffic much harder to track. Both reduce risk, but neither eliminates it if your device is compromised, if the VPN is subpoenaed, or if you log in to accounts over Tor (thereby revealing your identity to those sites).

### Rule 3: Use relevant threat models

Edward Snowden and the journalists who worked with him took extraordinary measures to safeguard themselves against **state-level actors** [7], but most of us aren’t involved in issues of national security and don’t need to take those kinds of precautions. Instead, we typically face 1 of 3 kinds of threat illustrated by the examples below.

- **Casual threats** are opportunistic. For example, Monica, a professor in psychology, is targeted by Mohan, an undergraduate in computer science who spends hours every day in online echo chambers complaining about how “SJW bullshit” is ruining tech. He really didn’t enjoy Monica’s guest lecture on discrimination and inclusivity in his software engineering class and thinks it would be a laugh to make her the target of anonymous abuse online. He is unlikely to invest significant effort in his attack (at least not initially), but his attack may be backed up by more knowledgeable members of online forums. They are almost certainly not computer security specialists; instead, they are probably older versions of Mohan who have picked up a few tricks and bits of software and enjoy the digital equivalent of throwing bricks through strangers’ windows.
- **Intimate threats** come from people who know their targets’ passwords or have a chance to install spyware on their targets’ devices [8]. For example, Elena, graduate student, is targeted by her former romantic partner Eric, who is also a graduate student but not in the same department. Their relationship had become increasingly abusive over the last 2 years. With the help of friends, Elena has moved out of their shared apartment and is rebuilding her life; Eric is obsessed with the idea that she left him for someone else and is now stalking her.
- **Insider threats** come from people who have legitimate access to accounts and devices. For example, Boris, professor of medicine, is targeted by Bethany, who works for the university’s IT department. Boris has agreed to serve as an expert witness in an upcoming liability case involving a large chemical company; Bethany has been asked by a former colleague to find out what he is going to say in order to discredit his testimony.

### Rule 4: Use a password manager

Using a weak password is a good way to ensure that your account will eventually be compromised, in part because **dictionary attacks** can be run offline against encrypted password files to find passwords that match common patterns. Using a clever password scheme, such as the name of the site plus a word only you know, does not increase security by much: Whatever scheme you have thought of, attackers have seen before. And since people are often identified on multiple sites by the same email address, as soon as 1 site where you’ve used that scheme is compromised, attackers can guess the scheme and use it elsewhere.

Reusing passwords ensures that damage spreads, so using a different password for each site helps limit harm if any are compromised. However, strong passwords are hard to remember and to type, so always use a **password manager** that generates strong passwords and saves them all under a master **passphrase**. Your passphrase should be several words long and something you are unlikely to forget. It does create a single point of attack, but is still safer than choosing passwords yourself, since password managers aren’t fooled by similar-seeming sites like paypaI.com.

Writing passwords down and keeping them in your wallet is not necessarily a bad practice—it depends on who is doing it. For example, an elderly person who finds tech confusing might well choose simple, easy-to-guess passwords for their accounts if they have to be remembered. On the other hand, they have a lifetime of practice keeping track of bits of paper, and will probably notice if their purse or wallet is stolen.

### Rule 5: Use two-factor authentication

Authentication relies on something you know (like a password), something you have (like a security key), or something you are (like your fingerprints). **Two-factor authentication** (2FA) requires two of these together to establish your identity, e.g., a password (which can be stolen electronically) plus a random code generated by an app on your phone (which means attackers need access to you).

2FA is as important to security as using a password manager, but where possible, you should rely on an app for 2FA instead of using text messages. What you should never do is share a confirmation code, since a common attack is to trigger a password reset and then call the victim pretending to be from the IT department and ask them to read the code back to “verify” your account. As soon as you do this, the attacker can change your password and get into your account.

Many security experts now recommend using a physical 2FA key such as a YubiKey, which fits on a keychain and plugs into a standard USB port. Sites like Tech Solidarity have easy-to-follow instructions explaining how to set them up for a range of popular social networking sites.

### Rule 6: Think before opening

Much of the software we use was designed in more innocent times, and since companies are almost never held liable for the damage caused by their software, they have consistently prioritized convenience for the many over harm to the few. One common example is documents that contain code called “macros” that automatically execute when the document is opened. Used for good, a macro can check that an address field has been filled in correctly. Used for evil, it can email everyone in your address book or send a copy of those addresses to anyone in the world. Microsoft Word and Excel are particularly notorious for this vulnerability, but many other kinds of documents have the same flaw.

Attempts to get you to open an email attachment, click on a link, install software, or log into a website are called **phishing** attacks. The strongest defense is to never do these things, but in the modern world, that would make most work impossible. The second-best defense is to take sensible precautions. If you are able, invest in virus scanning software such as Proofpoint to scan email attachments before you download them. While many email clients have virus scanning technology built-in, this will offer an extra layer of protection.

Similarly, don’t click links in emails without checking them first: Instead, hover over the link and see if it matches the site it claims to be. Alternatively, log into the site manually rather than following the provided link. It takes more time, but is still faster than fixing your credit rating. And when you go to a website, check the real domain name in the URL: paypaI.com with an upper-case “I” instead of a lower-case “l” is not the site it pretends to be, and wwwpaypal.com is a different domain than www.paypal.com.

Many sites send an email with a random URL to confirm your identity when you are resetting your password. On the one hand, this means that an attacker has to get access to your email in order to break into your account. On the other hand, random URLs are hard to type in, so these emails encourage us to click on links in emails. If you are not expecting a password reset email, don’t click on the link.

While phishing attacks are wide-ranging, **spear phishing** describes the use of data harvested from previous victims to attack specific targets. Here, the best defense is to verify suspicious emails, e.g., by phoning people to confirm their identity. It’s particularly important to do this when you are sent things like password reset instructions. Many IT departments send out messages that are indistinguishable from spear phishing attacks, which just trains people to be victims.

### Rule 7: Erase before discarding

Moving files into the trash and then emptying it does not actually erase the data: It just tells the computer that the space is available for reuse. (This is why reporters and private investigators regularly go dumpster diving.) The best way to address this problem is to encrypt your hard drive, which is a quick setup option for all major operating systems these days.

Even with that, you should act as if any device you throw away is going to fall into unfriendly hands. Use a secure deletion tool like BleachBit (Linux or Windows) or FileShredder (MacOS) before selling, recycling, or discarding your hardware, but keep in mind that this doesn’t affect backups or files stored online on sites like Dropbox. And keep in mind that it is practically impossible to truly delete data from social networking sites: In most cases, their “delete” usually means “don’t show any more” rather than “erase all past record of.”

### Rule 8: Check your devices and accounts periodically

Many tech companies who offer free products and services make money by selling targeted advertising to you using the data they have about you. They do give users some control over personal data, but they frequently change their terms of service in opaque ways. Seemingly innocuous information can give attackers valuable clues: Restaurant “likes” reveal where you were at specific times, while funny stories about childhood birthday parties reveal likely answers to security questions. Again, it’s a good practice to get into the habit of checking your privacy settings every time you do some other regular task.

Unfortunately, even if you do this, information may leak through other means. For example, attackers can friend your friends in an attempt to get information about you, such as the name of your first school. And as bad as social media sites are for social engineering in this way, cell phone applications are often worse (not even counting the ones that turn out to be government-sponsored spyware [9]). In general, if a game wants access to your camera and address book, you should probably find a different game to play.

Since social media is a fact of life for most of us, you should check your settings periodically, just as you would take your car in for an oil change. (The authors do these things at the same time in order to remember both.) Turn off everything you can, and then use a tracking blocker such as Ghostery to reduce information leakage.

Many experts recommend using separate devices or accounts for work and personal life, but this is increasingly unrealistic. Everyone checks their personal email from their work device eventually, and everyone uses their personal phone for 2FA. However, you should consider getting a second phone for international travel: The legalities around who can take your devices and/or force you to unlock them are complicated and frequently overstepped, so you should assume that anything on or connected to the devices you are traveling with will be compromised.

Never plug a random USB drive into your device: it’s like letting a complete stranger into your home unsupervised.

### Rule 9: Fight back

Casual attackers may eventually get bored and move on, but like all bullies, they will also often revisit previous victims, and even if they don’t, they are likely to pick new ones. If you have been attacked:

1. **Find support.** Being targeted is frightening and wearying, particularly if you belong to one of the many groups that are targeted in real life as well as online. Let family, friends, and colleagues know what is happening so that they can support you. They may also be able to offer advice if they have been in similar situations.
2. **Use anti-harassment apps** like Block Party and document everything. Save emails and take screenshots of sites like Facebook and Twitter (in case attackers delete or alter material).
3. **Do not engage directly.** Casual attackers are often seeking attention, so a direct response often encourages further attacks (and can draw attention from like-minded attackers).
4. **Report the attack.** Social media sites have done everything they can to avoid legal accountability for online attacks, but companies and universities will usually take what steps they can once they know there is a problem. In the authors’ experience, they are more inclined to take real action against the attacker if they believe that you might speak publicly about what has happened and thereby damage their reputation, so never agree to a nondisclosure agreement that would prevent you from doing so.

### Rule 10: It’s not all about you

Our final rule brings us full circle to the first one. We don’t just wear masks to prevent ourselves from becoming infected: We also wear them so that we will not infect others. Similarly, if you do not take precautions with online security, then you are putting others at risk. Simple steps like putting passwords on PDFs that contain sensitive information can go a long way to deter attackers, in the same way that a sturdy-looking bike lock encourages would-be thieves to go after some other bike. And if you are compromised, let those affected know as soon as you can.

The only long-term way to improve everyone’s online safety is to pressure politicians to strengthen liability legislation so that companies, universities, and other institutions have real incentives to take meaningful action. Cars and drugs are as safe as they are because their manufacturers are liable for negligence and harm. The sooner software companies and social media sites are liable as well, the safer all of us will be.
